# Possibilities of Controlling the Quantum States of Hole Qubits in an Ultrathin Germanium Layer Using a Magnetic Substrate: Results from ab Initio Calculations

**DOI:** 10.3390/nano13233070

**Published:** 2023-12-03

**Authors:** Andrey N. Chibisov, Mary A. Chibisova, Anastasiia V. Prokhorenko, Kirill V. Obrazcov, Aleksandr S. Fedorov, Yang-Xin Yu

**Affiliations:** 1 Computing Center, Far Eastern Branch of the Russian Academy of Sciences, Khabarovsk 680000, Russia; 2 Kirensky Institute of Physics, Federal Research Center KSC SB RAS, Krasnoyarsk 660036, Russia; 3 Laboratory of Chemical Engineering Thermodynamics, Department of Chemical Engineering, Tsinghua University, Beijing 100084, China

**Keywords:** density functional theory, quantum state, hole qubit, electronic structure, electric field

## Abstract

Using density functional theory in the noncollinear approximation, the behavior of quantum states of hole qubits in a Ge/Co:ZnO system was studied in this work. A detailed analysis of the electronic structure and the distribution of total charge density and hole states was carried out. It was shown that in the presence of holes, the energetically more favorable quantum state is the state |0˃, in contrast to the state |1˃ when there is no hole in the system. The favorability of hole states was found to be dependent on the polarity of the applied electric field.

## 1. Introduction

The problem of creating a quantum transistor that operates on the new principles of hole qubits and performs quantum operations without errors is the most pressing task for modern nanoelectronics [1,2,3]. The construction of such a transistor will make it possible to design a quantum processor with high computing power that is capable of solving important scientific problems in a short time in various fields such as materials science, medicine, and machine learning. The most promising material for creating quantum computers is flat germanium nanowire [4,5,6], due to the fact that the holes in this nanowire have a strong spin–orbit coupling [7], a light effective mass [8], ease of control of the electric field [9], compatibility with existing Si-technology, and ability to operate in much lower magnetic fields [10]. In Ref. [11], it was shown that excited hole levels make a significant contribution to the g factor and its derivative with respect to the electric field. Moreover, the spin–hole qubits approach sometimes exceeds the performance of qubits with electron spin [12].

To date, the problem of controlling hole qubits to obtain precise logical operations or their correction remains technologically challenging. This is because the process of controlling hole qubits’ quantum states via a magnetic field is associated with the complexity of constructing a special installation in which this magnetic field is created. In this work, we theoretically demonstrate for the first time the use of a new ultrathin two-dimensional Co:ZnO magnets [13,14,15] as a magnetic substrate to create external magnetization, which creates a magnetic field in the germanium structure and, accordingly, influences hole qubits with the ability to control them. This study analyzes the advantage of hole states depending on the applied electric and magnetic fields.

## 2. Methods and Details of Calculations

Calculations of the equilibrium structures, as well as electronic and magnetic properties, were performed by means of the VASP package [16,17,18]. A generalized gradient approximation in the GGA–PBE form [19] in the PAW pseudopotentials [20,21] was used for estimating the exchange-correlation potential. The noncollinear calculations [22] were performed, taking into account spin–orbit coupling [23] with additional GGA + U corrections [24]. The effective on-site Coulomb interactions were set as equal to the following values: U_p_(Ge) = 2.0 eV, U_d_(Zn) = 10.5 eV, U_p_(O) = 7.0 eV, and U_d_(Co) = 3.3 eV. To calculate the magnetic properties of atomic systems, first the initial magnetic moment was set for each atom, then complete relaxation of the atomic system was carried out taking into account the generalized local-spin-density theory [22] and the resulting magnetization on the atoms was determined from the results obtained. For multi-layered systems, van der Waals corrections were used based on the semiempirical Grimme’s DFT-D3 method with the Becke–Johnson damping function [25]. We used the plane–wave basis with a cutoff energy of 600 eV. All slab calculations were performed with a 6 × 6 × 1 k-point set using the Monkhorst–Pack scheme [26]. To test the bulk germanium unit cell, we used an 18 × 18 × 18 k-point set. The optimization of the atomic structure continued until the forces acting on atoms became less than 0.01 eV/Å.

## 3. Results and Discussion

**Figure 1:**
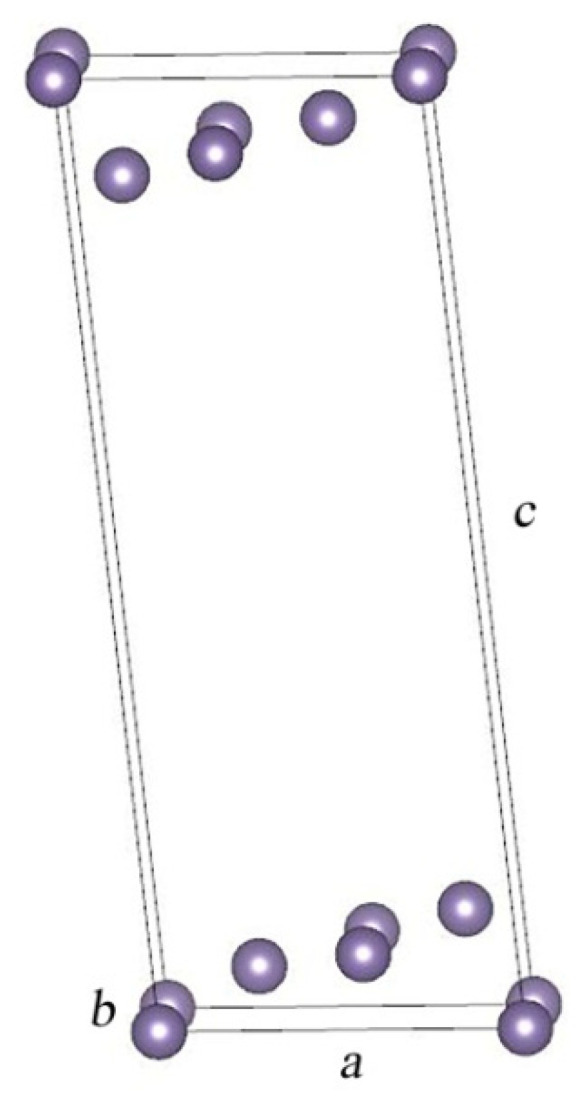
Germanium slab as a model layer for a hut wire.

Previously, in our work [4], we proposed a 2D structural model with the most stableGe {105} surfaces for germanium hut wires. It was shown that for this model, with the relaxation of atoms, it is more advantageous to exist with monoclinic symmetry in the space group P2/m. The smallest structural basis with which this two-dimensional layer can be built is presented in Figure 1. It is characterized by the space group P2/m and has four Ge atoms in its structure located at the following atomic positions within the point symmetry group C_2h_: two 2n positions with symmetry m, one 2m position with m symmetry, and another 1a position with 2/m symmetry. The cell parameters are equal to the following values: *a* = 6.3060 Å, *b* = 4.2808 Å, and *c* = 18.2268 Å, with the angles equal to *α* = 89.87°, *β* = 115.67°, and *γ* = 89.99°, respectively.

**Figure 2:**
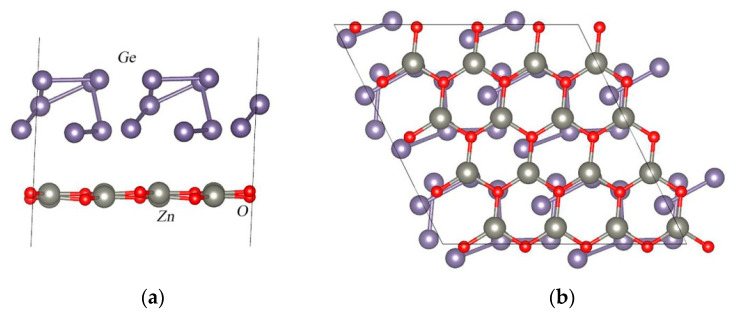
Equilibrium atomic structure for the Ge/ZnO interface: (**a**) side view and (**b**) bottom view.

**Figure 3:**
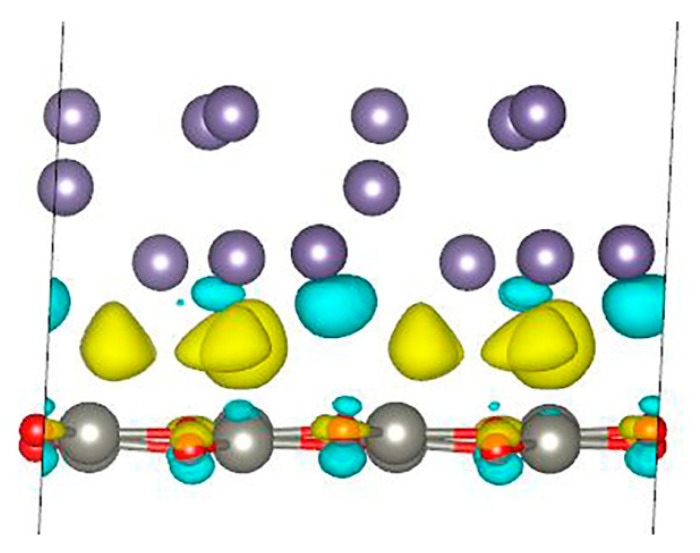
Charge density difference for Ge/ZnO interface. Yellow color indicates places where charge increases, and turquoise color indicates places where charge decreases.

A two-dimensional monolayer of zinc oxide with an increased cell size of 4 × 4 × 1 was combined with a germanium slab with a size of 2 × 2 × 1 (Figure 2). As a result, the supercell with the Ge_28_/Zn_16_O_16_ composition was obtained. Then, the complete structural relaxation of this system was carried out. In the equilibrium structure, the distance between the Ge and ZnO surfaces was 3.079 Å. Figure 2 shows the equilibrium atomic structure for the Ge/ZnO interface. Next, we calculated the binding energy of the two-dimensional germanium and the 2D ZnO structure per unit area using the following equation:

$$E_{b}=\left(E_{Ge\_ZnO}-E_{ZnO}-E_{Ge}\right)/S,$$

where *E_Ge_ZnO_* is the total energy of the Ge/ZnO interface, *E_ZnO_* is the total energy of the 2D ZnO structure, *E_Ge_* is the total energy of the germanium structure, and *S* is the Ge/ZnO surface area. The calculations show that the binding energy is −0.192 eV/Å^2^ per unit surface area. Thus, this system can be obtained experimentally. To analyze the charge distribution, the difference in charge density for the Ge/ZnO interface was calculated (Figure 3). An analysis of the results shows that during the formation of the Ge/ZnO structure, electrons flow mainly from the lower layer of germanium atoms and partially from oxygen atoms and are localized mainly in the interlayer space between the Ge and ZnO layers. Moreover, the charge on zinc atoms and germanium atoms (except for those in the lower layer) practically does not change, but on oxygen atoms, the charge is polarized. The calculations show that the germanium layer transfers an additional charge of 0.218 *e* to the ZnO layer. Thus, the germanium slab is a donor for the ZnO layer.

**Figure 4:**
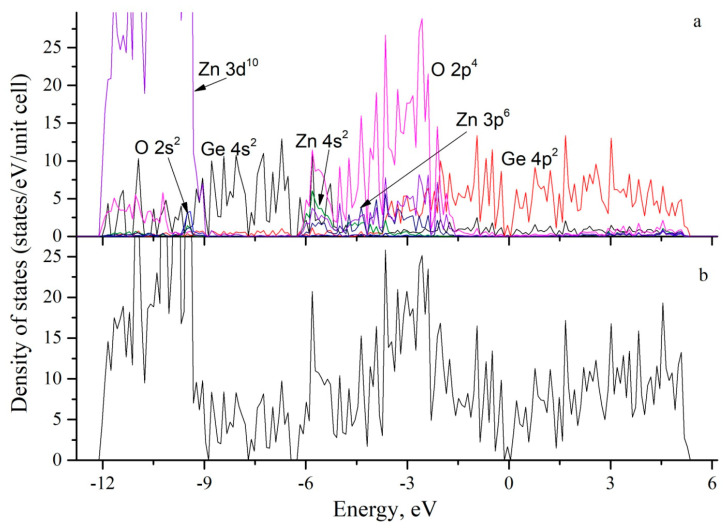
Calculated densities of states for the Ge/ZnO interface: (**a**) partial densities of states and (**b**) total densities of states.

To determine the nature of the interaction of atoms in the Ge/ZnO system, the total and partial electron densities of states were calculated. The total electron density of states (Figure 4b) shows that the band gap for the pure Ge/ZnO compound is 0.13 eV. This value is much less than for bulk zinc oxide, which is 3.30 eV (Table 1), as calculated using the GGA + U method based on the ATK code [27], and less than 3.40 eV, as obtained using the VASP code [28]. The value obtained from the experimental data is 3.37 eV [29]. Our calculation for pure bulk zinc oxide gives a band gap of 3.29 eV. For the two-dimensional layer of zinc oxide, with one-atom thickness, the band gap is 3.04 eV, according to our calculations. This value is in good agreement with the value of 3.28 eV [30] obtained by using the Heyd–Scuseria–Ernzerhof (HSE06) hybrid functional method. In Table 1, the calculated band gaps for the monolayer, bulk zinc oxide, and Ge/ZnO and Ge/Co:ZnO compounds are presented.

Figure 4a shows the partial densities of states for Ge, Zn, and O atoms during the formation of the Ge/ZnO structure. It can be seen that the binding of the germanium slab and the two-dimensional zinc oxide layer occurs due to the hybridization of the Ge 4p^2^–Zn 3d^10^–Zn 4s^2^, Ge 4p^2^–O 2p^2^, and Ge 4s^2^–O 4p^4^ orbitals. From the density of states shown in Figure 4 for Ge/ZnO, it can be seen that the top of the valence band and the bottom of the conduction band are determined by the 4p^2^ states of germanium.

Next, we studied the effect of impurity due to cobalt atoms on the atomic structure and electronic properties of the Ge/Co:ZnO system. In Ref. [13], Rui Chen et al. showed that cobalt atoms replace zinc atoms in a concentration of 1 Co: 7 Zn, and at this concentration, the maximum magnetization in the material is achieved with a random arrangement of cobalt atoms in the zinc oxide layer. In our case, we investigated this cobalt concentration at the Ge/Co:ZnO interface. The calculation results show that the band gap for Ge/Co:ZnO is also 0.13 eV, as for Ge/ZnO (Table 1). Thus, the band gap does not change with the introduction of a cobalt atom due to the fact that the top of the valence band and the bottom of the conduction band are completely determined by the 4p^2^ states of germanium.

**Figure 5:**
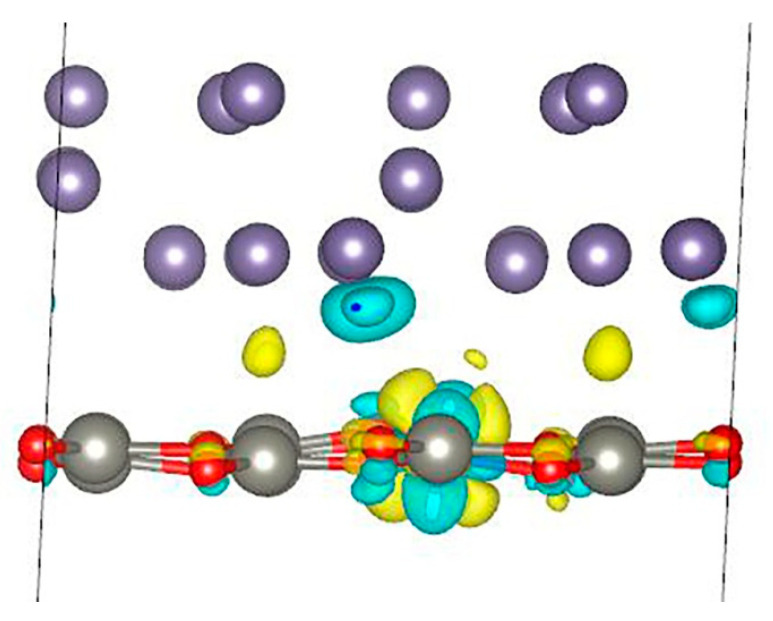
Charge density difference for Ge/Co:ZnO interface.

Next, we calculated the charge density difference for the Ge/Co:ZnO interface (Figure 5). The results show that during the formation of the Ge/Co:ZnO structure, a redistribution of electrons occurs in the d_xz_ and d_yz_ orbitals of cobalt atoms. Electrons also flow from the lower layer of germanium atoms and partially from oxygen atoms and are localized in the interlayer space between the Ge and ZnO layers. The calculations of charges on atoms using the Bader method [31] show that, in this case, the germanium layer transfers an additional charge of 0.213 e to the Co:ZnO layer. This charge value of 0.005 e is less than in the absence of cobalt in the zinc oxide structure. Thus, it is clear that the introduction of cobalt into zinc oxide leads to a decrease in the charge transferred to the ZnO layer.

**Figure 6:**
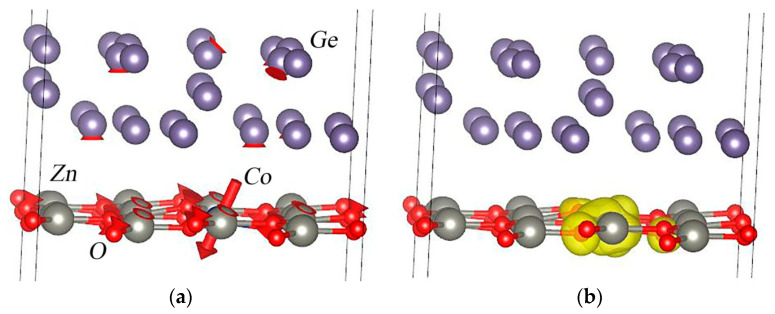
Equilibrium magnetic properties for the Ge/Co:ZnO structure: (**a**) direction of magnetic moments on the atoms and (**b**) spin density around a cobalt atom and its nearest oxygen atoms.

Next, we studied the magnetic properties that arise in the system when a cobalt atom is introduced into a thin ZnO layer (Figure 6). In this case, two magnetic states were studied, with spin “up” and “down”. The calculations show that the spin-down state is the most favorable at 5 meV. In this state, the magnitude of the magnetic moment on the cobalt atom is 2.65 μB, with coordinates in the Bloch sphere equal to (θ; φ) = 140.26°; −37.29°. Thus, the magnetic moment on the cobalt atom lies in the second half of the Bloch sphere and corresponds to the quantum state |1˃. The total magnetic moment of the system is 3.00 μB in the same quantum state.

**Figure 7:**
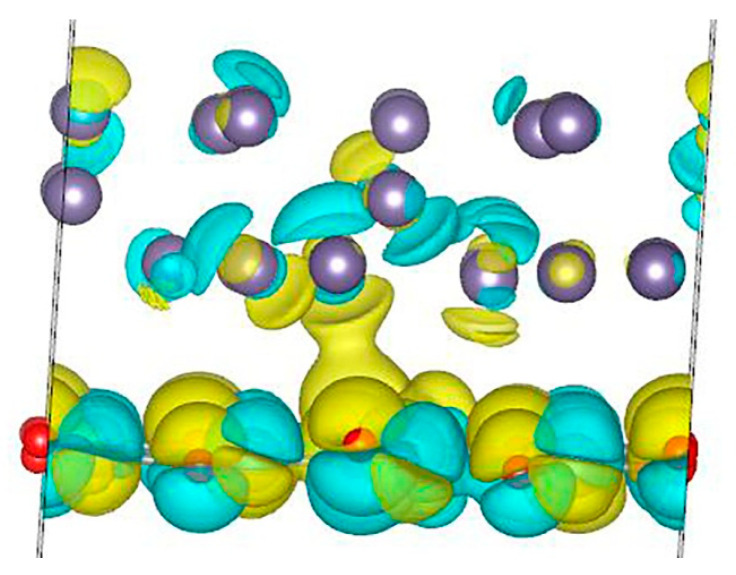
Localization of hole states for one hole in the Ge/Co:ZnO structure.

Next, we calculated the localization of hole states in a Ge/Co:ZnO system containing cobalt. The calculations show that hole states are localized mainly in the germanium layer and partially in the zinc oxide layer (Figure 7). Indeed, the calculation of atomic charges shows that in the Ge/Co:ZnO system without a hole, the total charge is 111.787 e on the Ge layer and 285.213 e on the Co:ZnO layer. When a hole is formed, the charge on the layers changes to be 110.948 e for the Ge layer and 285.052 e for the Co:ZnO layer. Thus, the calculation clearly shows that it is the germanium layer that loses a larger portion of charges, and this value is 0.839 e. Due to the presence of excess positive charges in the system, electron polarization occurs in the ZnO layer. The calculations show that the spin-up magnetic state is the most favorable, in this case, at 0.5 meV. Moreover, the total magnetic moment of the entire system in a given magnetic state increases and amounts to 3.16 μB, and in the Bloch sphere, its coordinates are equal to (θ; φ) = 1.98°; −31.50°. The magnetic moment on a cobalt atom is 2.66 μB in the same magnetic quantum state |0˃ in the lower hemisphere of the Bloch sphere. Thus, we can conclude that in the presence of a hole in the Ge/Co:ZnO system, the energetically more favorable quantum state is the state |0˃, in contrast to the state |1˃ when there is no hole in the system. Thus, it is clear that in this case, the total magnetic moment of the system and the magnetic moment on the cobalt atom are almost 10 times easier to transfer from one quantum state to another. In this case, you need to spend only 0.5 meV, in contrast to the case when there are no holes in the system (which needs 5 meV).

Next, we investigated the influence of the electric field on the process of transition between quantum states |0˃ and |1˃. We calculated the values of the total energies of the system depending on the magnitude and direction of the applied field. The calculations show that with an applied field equal to −0.01 eV/Å, for the transition between quantum states |0˃ and |1˃, it is necessary to expend energy equal to 134 meV. For a field equal to +0.01 eV/Å, the transition between states |0˃ and |1˃ occurs with the expenditure of energy equal to only 5 meV. Thus, it is clear that the application of a negative electric field prevents the transition between quantum states |0˃ and |1˃, and a positive electric field promotes this transition. This occurs due to the violation of inversion symmetry and the appearance of new non-degenerate states in the material under the influence of the magnetic field [32,33,34,35,36].

## 4. Conclusions

In this work, quantum mechanical calculations were carried out to investigate the behavior of atomic magnetic moments in a Ge/Co:ZnO system in the presence or absence of a hole qubit. The results show that the germanium layer is a donor of charge for the ZnO layer, and the introduction of cobalt into the zinc oxide layer leads to a decrease in the charge transferred to the ZnO layer. A detailed analysis of the electronic structure shows that the band gap for the pure Ge/ZnO compound is 0.13 eV. The introduction of a cobalt atom does not lead to a change in the band gap for Ge/Co:ZnO due to the fact that the top of the valence band and the bottom of the conduction band are determined by the 4p^2^ states of germanium. Moreover, the spin-down state is the most favorable at 5 meV for the Ge/Co:ZnO system. It is revealed that hole states are localized mainly in the germanium layer and partially in the zinc oxide layer. In the presence of a hole in the Ge/Co:ZnO system, the energetically more favorable quantum state is the |0˃ state, in contrast to the |1˃ state when there is no hole in the system. In this case, the total magnetic moment of the system and the magnetic moment on cobalt atoms are easier to transfer from one quantum state to another, in contrast to the case when there are no holes in the system. It is shown that a negative electric field prevents the transition between quantum states |0˃ and |1˃, and a positive electric field promotes this transition. This occurs due to the violation of inversion symmetry and the appearance of new non-degenerate states in the material under the influence of the magnetic field.

## Figures and Tables

**Table 1:** The electronic energy gap in Ge/ZnO according to the GGA + U method.

Structure	Energy Gap	Ref.
monolayer ZnO	3.04	3.28 [30]
bulk ZnO	3.29	3.30 [27] 3.40 [28] 3.37, Exp. [29]
Ge/ZnO	0.13	—
Ge/Co:ZnO	0.13	—

## Data Availability

Data are contained within the article.
